# Supplementary material for: Natural variation in CTB4a enhances rice adaptation to cold habitats
Source: Nat Commun. 2017 Mar 23;8:14788. doi: 10.1038/ncomms14788 (PMC5376651; doi:10.1038/ncomms14788)
Supplement: Supplementary Information — Supplementary Figures and Supplementary Tables [file ncomms14788-s1.pdf]

Supplementary information

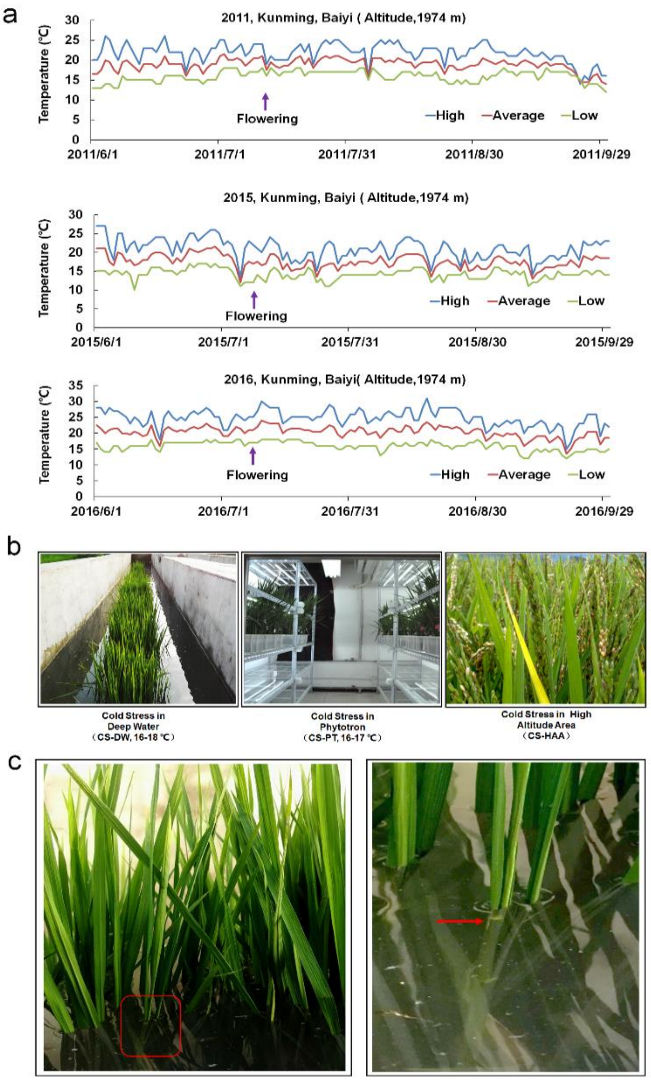

Supplementary Figure 1. Cold stress conditions used in this study. (a) Weather records of Baiyi township in Kunming city in the summers of 2011, 2015 and 2016 obtained from the local weather station. (b) Three kinds of cold stress conditions. CS-DW, cold deep water from a well was used for cold stress. CS-PT, plants were transplanted in pots and allowed to recover for two weeks before cold stress in a phytotron. CS-HAA, the materials were planted at Baiyi township in Kunming city where the average temperature during the rice growing period was below <20 ℃. (c) The panicles at the booting stage specifically referring to the meiosis stage of the pollen mother cell were treated under CS-DW and CS-PT. The leaf sheath of the flag leaf is just extracted from that of the penultimate leaf as marked with red box in the left picture and red arrow in the right picture.

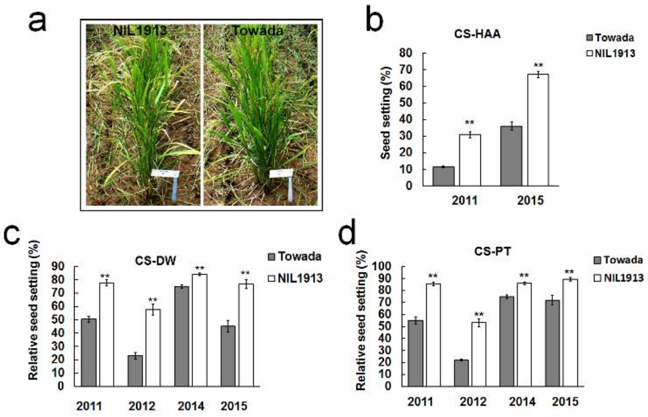

Supplementary Figure 2. Characterization of NIL1913 under three cold stress conditions over several years. (**a**) Phenotypes of NIL1913 and Towada under CS-HAA. NIL1913 had more filled grain number than Towada. **(b)** Seed setting of NIL1913 and Towada under CS-HAA. (**c, d**) Relative seed setting of NIL1913 and Towada under CS-DW (**c**) or CS-PT (**d**) over several years. Data represent means ± s.d. (n=15) in C, D and E, **P < 0.01, Student's t test.

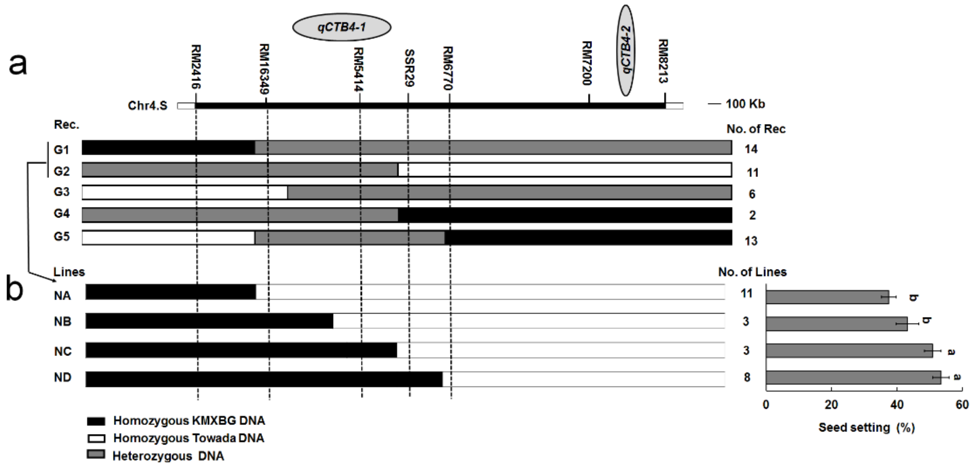

Supplementary Figure 3. Primary mapping of *CTB4a.* G1–G5 represent the five kinds of recombinant individuals. Twenty five recombinants of G1 and G2 without *qCTB4-2* were identified as NA, NB, NC or ND for analysis.

a

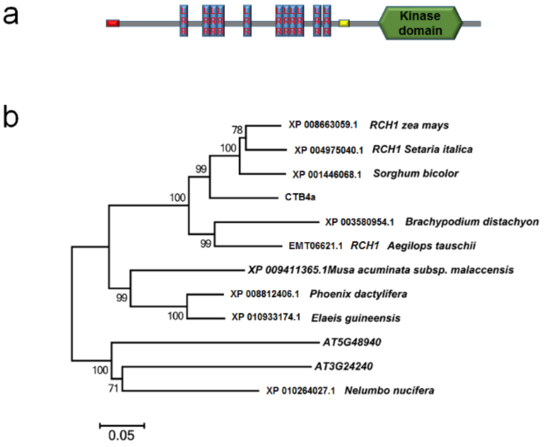

c

Percent Identity (upper right); Divergence (lower left)

	1	2	3	4	5	6	7	8	9	10	11	12	13		
1		89.7	86.2	80.7	80.8	72.4	65.4	66.8	67.7	67.6	56.3	55.0	58.5	1	XP 008663059.1
2	11.1		86.1	80.5	80.4	72.8	65.4	67.2	68.2	68.0	55.9	55.8	59.4	2	XP_004975040.1
3	15.3	15.4		79.7	79.6	72.1	65.1	64.1	65.6	65.4	55.6	55.2	56.8	3	XP_002446068.1
4	22.4	22.7	23.8		99.8	74.8	66.0	65.9	67.8	67.1	56.3	55.2	58.3	4	CTB4a-KMXBG
5	22.2	22.8	23.9	0.2		74.9	66.1	65.9	67.8	67.1	56.3	55.1	58.4	5	CTB4a-Towada
6	34.4	33.7	34.9	30.8	30.6		69.7	62.2	64.7	64.3	55.1	55.5	57.9	6	XP_003580954.1
7	46.3	46.1	46.7	45.1	44.9	38.8		54.6	57.2	57.4	46.8	47.5	49.2	7	EMT06621.1
8	43.7	42.9	48.6	45.2	45.2	52.2	68.5		75.7	75.4	57.5	57.3	61.5	8	XP_009411365.1
9	42.1	41.3	45.8	42.0	42.0	47.4	62.6	29.4		92.4	58.7	59.3	63.7	9	XP_008812406.1
10	42.2	41.7	46.3	43.2	43.2	48.2	62.0	29.9	8.0		58.7	58.9	63.7	10	XP_010933174.1
11	64.6	65.5	66.0	64.6	64.6	67.2	88.7	61.9	59.3	59.3		62.2	65.4	11	AT5G48940
12	67.5	65.7	67.0	67.0	67.2	66.2	86.8	62.2	58.1	58.7	52.2		67.3	12	AT3G24240
13	59.6	57.7	63.3	60.1	60.0	61.0	82.0	53.5	49.4	49.4	46.3	42.8		13	XP_010264027.1
	1	2	3	4	5	6	7	8	9	10	11	12	13		

Supplementary Figure 4. ***CTB4a* encodes a conserved LRR-RLK.** (**a**) Schematic diagram of CTB4a protein. The signal peptide and transmembrane region are indicated by red and yellow box respectively. (**b**) Phylogenetic analysis of CTB4a by MEGA 5.0 software. Amino acid sequences were downloaded from Uniprot (http://www.uniprot.org/). The scale bar represents 5% estimated sequence divergence. (**c**) Amino acid identity analysis of CTB4a in different species was conducted by Clustal W in DNASTAR-MegAlign software.

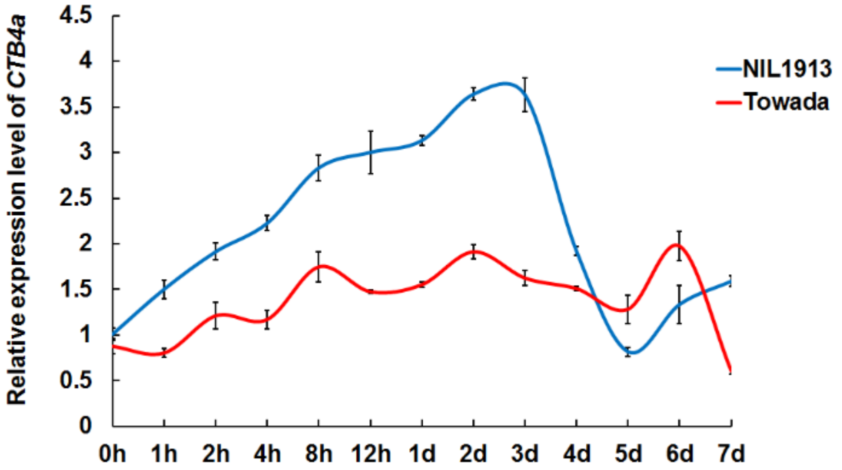

Supplementary Figure 5. qRT-PCR analysis using total RNA isolated from the leaves of NIL1913 and Towada under CS-PT at different time points. Leaf tissues stressed under CS-PT were sampled for RNA preparation. Three biological RNA samples were prepared and each experiment was performed with three technical repeats. Data represent means ± s.d. (n=3).

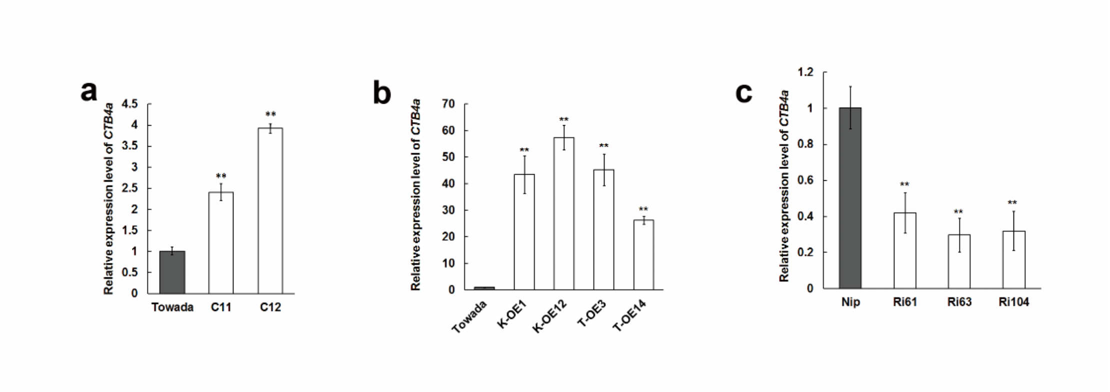

Supplementary Figure 6. Expression of *CTB4a* in transgenic lines. (**a**) $CTB4a^{KMXBG}$ complementation lines C11 and C12. (**b**) *CTB4a* overexpressing lines K-OE1, K-OE12, T-OE3 and T-OE14. (**c**) *CTB4a* RNAi lines. At least three independent RNA samples were prepared and each experiment was performed with three technical repeats. Data represent means ± s.d. **P < 0.01, Student's t test.

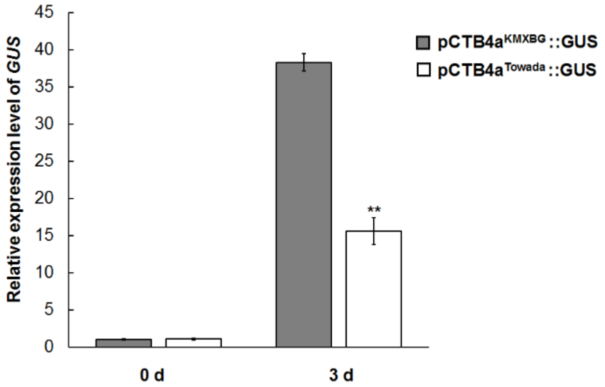

Supplementary Figure 7. Promoter activity analysis. Transgenic lines with single copy of pCTB4a^{KMXBG}::GUS or pCTB4a^{Towada}::GUS were isolated and subjected to cold stress. At least three RNA samples were prepared and each experiment was performed with three technical repeats. Data represent means ± s.d. (n=3). **P < 0.01, Student's t test.

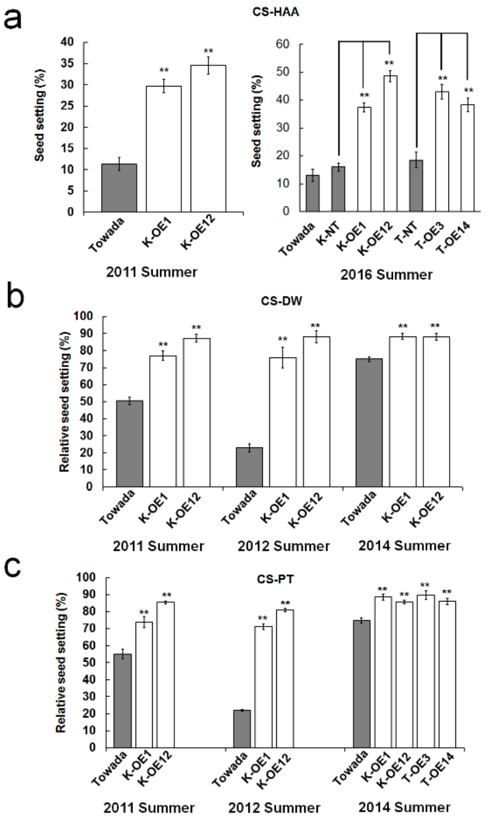

Supplementary Figure 8. Over expression of *CTB4a* enhanced cold tolerance in rice at the booting stage under different cold stress conditions over several years. Statistical results for seed setting of Towada and overexpression lines under CS-HAA (**a**), CS-DW (**b**) and CS-PT(**c**). Data represent means ± s.d. (n=20). K-NT and T-NT indicates the non-transgenic plants. **P < 0.01, Student's t test.

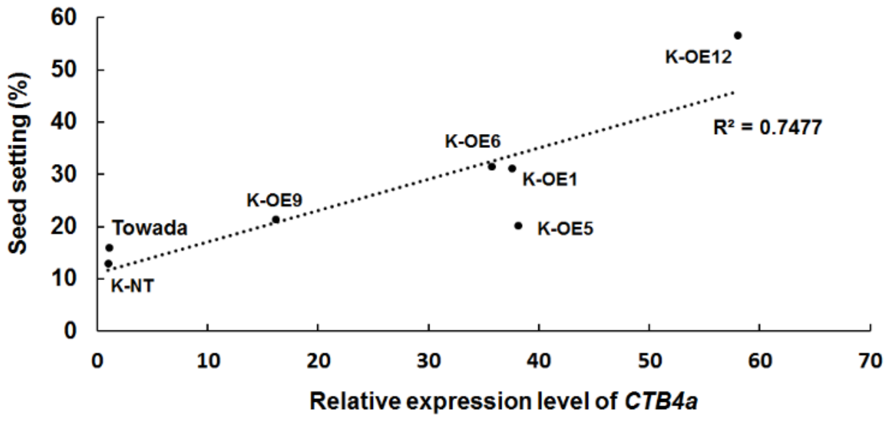

Supplementary Figure 9. Linear regression of seed setting and relative expression levels of *CTB4a* in different over expression lines. Statistical results for seed setting of Towada and overexpression lines under CS-HAA.

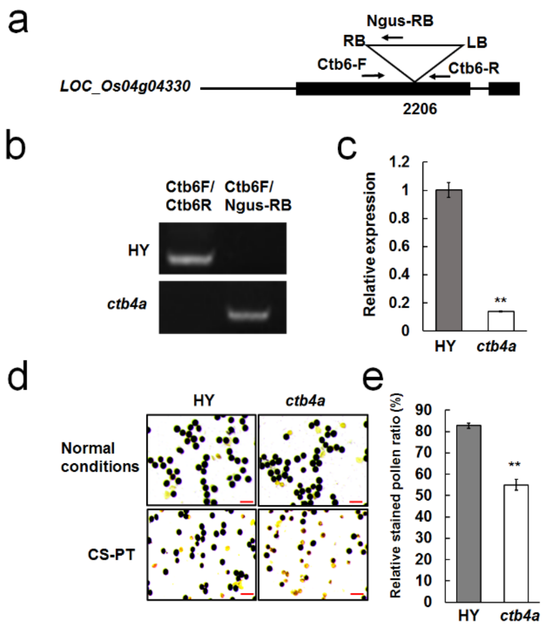

Supplementary Figure 10. *ctb4a* is sensitive to cold stress at the booting stage. (**a**) Diagram in the *ctb4a* mutant gene with a T-DNA insertion in the first exon of *CTB4a* at +2209 bp downstream from the ATG in HY background. Unfilled triangular box indicates the insertion location. RB, right border. LB, Left border. Primers marked with arrows are provided in Supplementary Table 6. (**b**) PCR identification of *ctb4a* using genomic DNA. (**c**) Expression levels of *CTB4a* in HY and the *ctb4a*. At least three RNA samples were prepared and each experiment was performed with three technical repeats. Data represent means ± s.d. (n=3) **P < 0.01, Student's t test. (**d**) Pollen fertilities shown by I_2-KI staining. Scale bars, 50μm. (**e**)The blue stained pollen grains were counted for calculating pollen fertility. Data represent means ± s.d. (n=10), **P < 0.01, Student's t test. Three spikelets were examined for each individual.

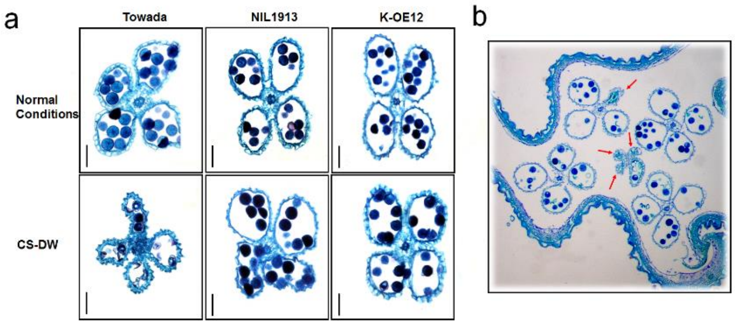

c

	Anther chamber	Towada	NIL1913	K-OE1	K-OE12	T-OE3
Normal conditions	Abnormal	16 (4.17%)	24 (6.19%)	31 (9.45%)	15 (5.43%)	23 (7.77%)
	Normal	368 (95.83%)	364 (93.81)	297 (90.55%)	261 (94.57%)	273 (92.23%)
	Total	384	388	328	276	296
CS-DW	Abnormal	107 (34.74%)	82 (24.40%)	68 (23.61%)	68 (22.97%)	75 (21.80%)
	Normal	201 (65.26%)	254 (75.60%)	220 (76.39%)	228 (77.03%)	269 (78.20%)
	Total	308	336	288	296	344

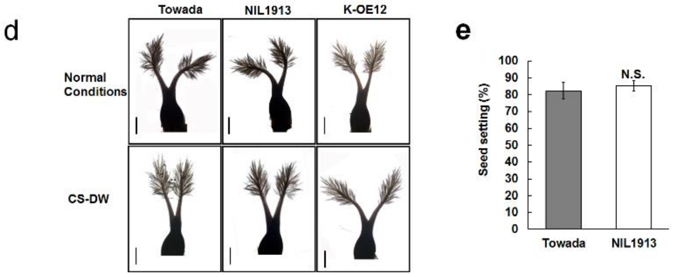

Supplementary Figure 11. Morphological changes of the anther and the pistil in Towada, NIL1913 and *CTB4a* overexpression lines under normal and CS-DW. (a) Microscopic observation of the anther in Towada, NIL1913 and *CTB4a* overexpression line. Scale bar, 50 μm. **(b, c)** Parts of the distorted pollen chamber adhered into pieces and reduced fertile pollen grains. The statistical results were listed in **(c).** (d) Pistil development in Towada, NIL1913 and *CTB4a* overexpression line. Pistil development was unaffected. Scale bar, 0.5 mm. **(e)** Seed setting showed no significant difference between cold stressed Towada and NIL1913 plants following hand-pollination from non-stressed NIL1913 plants. Data represent means ± s.d. (n=8). P values were calculated by Student's t tests.

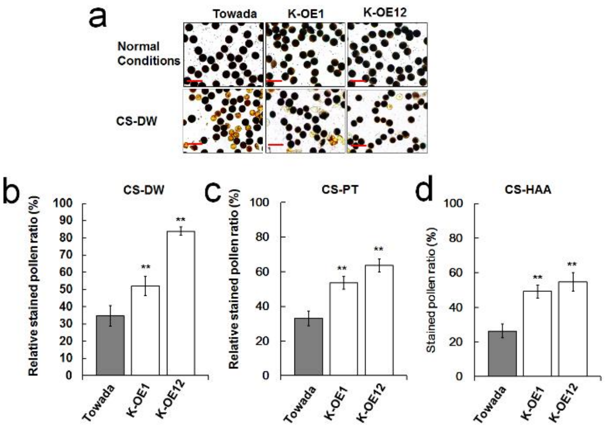

Supplementary Figure 12. Pollen fertility of Towada and *CTB4a* overexpression lines under cold stress. (**a**) Pollen fertility evaluated by I_2-KI staining. Blue stained pollen grains were counted for calculating pollen fertility of samples collected from plants grown under CS-DW (**b**), CS-PT (**c**) and CS-HAA (**d**) conditions. Data represent means ± s.d. (n=10). Three spikelet were examined for each individual. P values were calculated by Student's t tests, **P < 0.01.

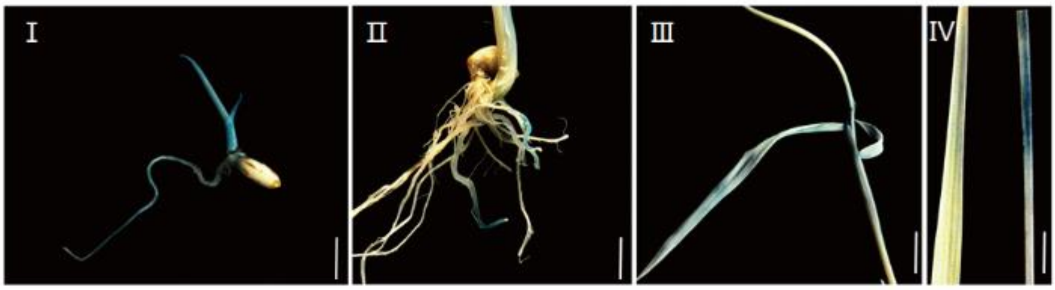

Supplementary Figure 13. Expression of *CTB4a* at the vegetative stage. Ⅰ, buds. Ⅱ, roots. Ⅲ, seedlings. Ⅳ, leaves and leaf sheaths. Scale bar, 1 cm in Ⅰ and Ⅱ, 0.5 cm in Ⅲ and Ⅳ.

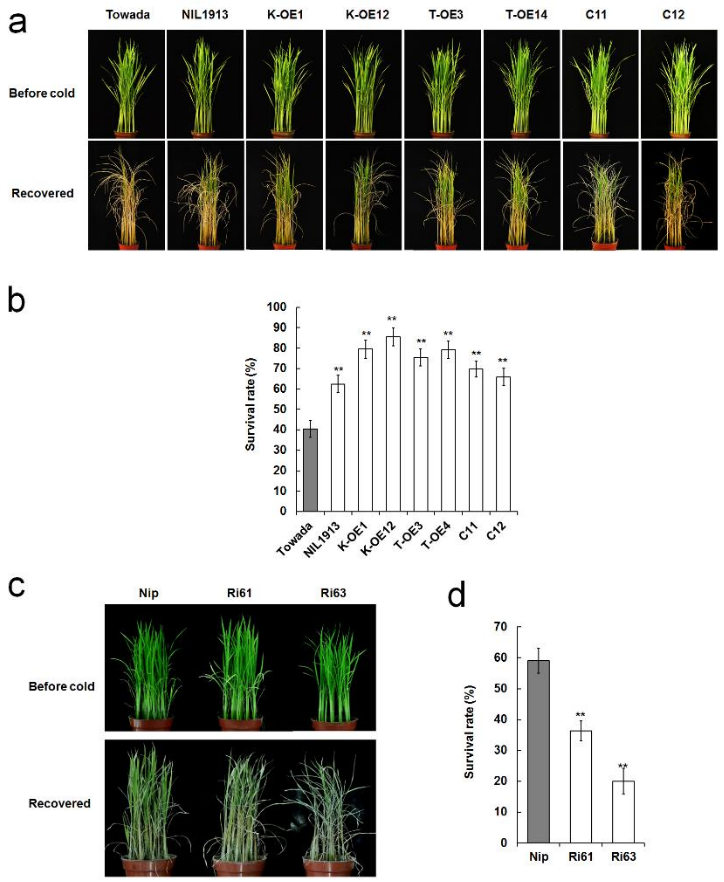

Supplementary Figure 14. *CTB4a* is essential for chilling tolerance at the vegetative stage. Chilling tolerance evaluation of Towada, NIL1913, overexpression and complementation lines (**a**), and RNAi lines (**b**). Upper, photographed before cold stress. Lower, photographed after cold stress (4-5 ℃) for 7 days and recovery for 10 days. Individuals with fresh green leaves were counted to determination of survival rates (**c, d**). Each experiment was involved at least 5 pots, each with 20 plants. Data represent means ± s.d. (n=5). All P values were calculated by Student's t tests. **P < 0.01.

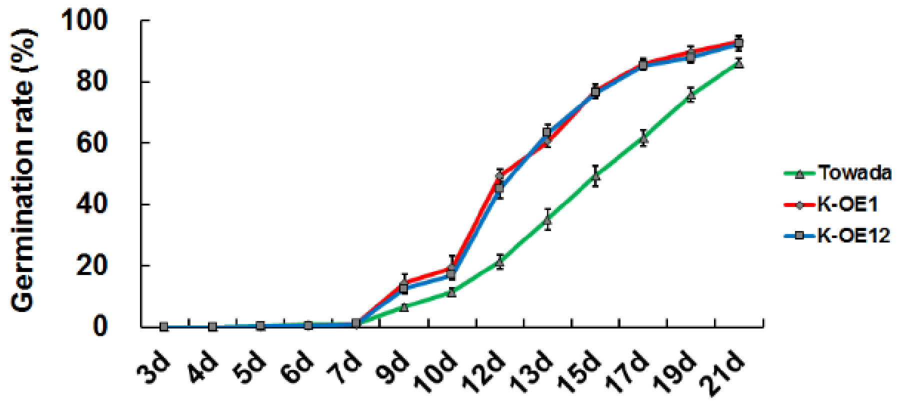

Supplementary Figure 15. Germination rates of Towada and *CTB4a* overexpression lines under cold stress. Surface-sterilized seeds were grown at 16 ℃ in 9 cm Petri dish and germinated seeds were counted. Each experiment consisted of at least 3 dishes, and each with 50 seeds. Data represent means ± s.d. (n=3).

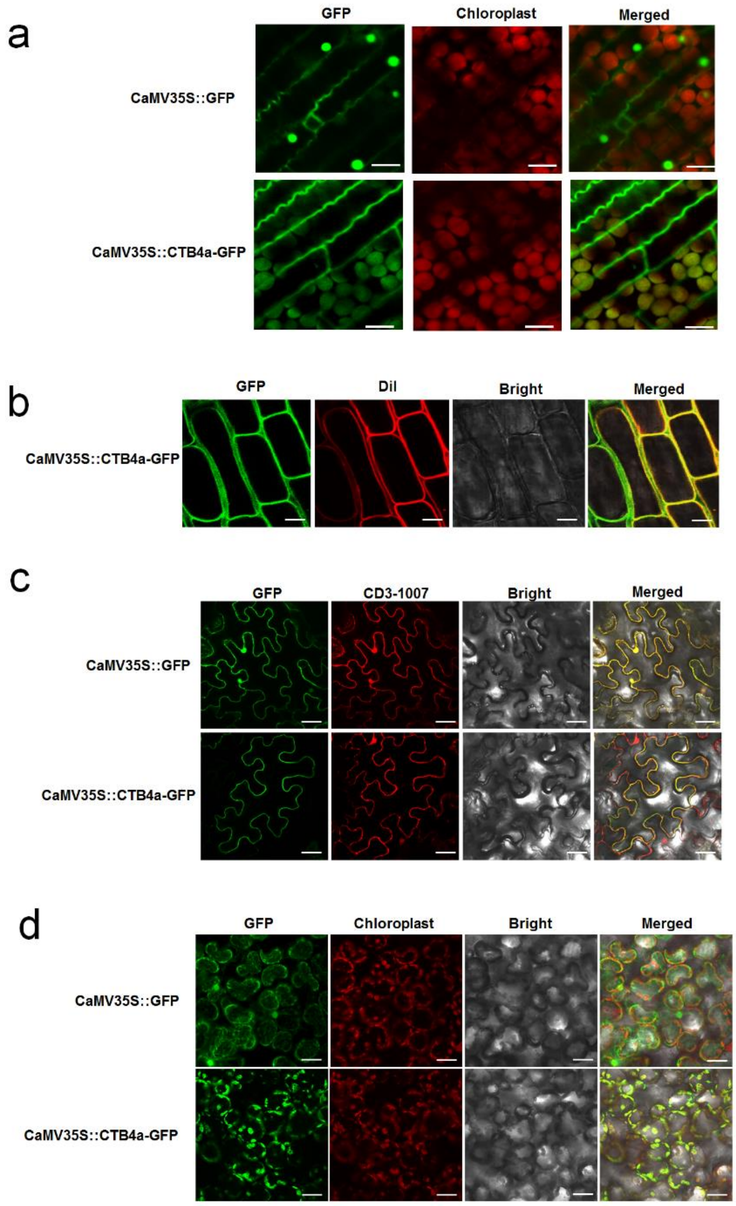

Supplementary Figure 16. Subcellular localization of CTB4a. (**a**) Leaf sheaths of transgenic rice lines were observed directly by confocal laser scanning microscopy. Scale bar, 10 μm. (**b**) Roots of CaMV35S::CTB4a-GFP rice transgenic lines were stained with Dil at 37 ℃ for 30 min and washed with PBS buffer three times for observation. Scale bar, 10 μm. (**c**) Co-localization of CTB4a-GFP with the red fluorescent plasma lemma marker pm-rk-CD3-1007 in tobacco epidermal cells. CTB4a-GFP overlapped with CD3-1007 at the plasma membrane. Scale bar, 40 μm. (**d**) CTB4a-GFP signal overlapped with spontaneous red fluorescence of chloroplasts. Scale bar, 30 μm.

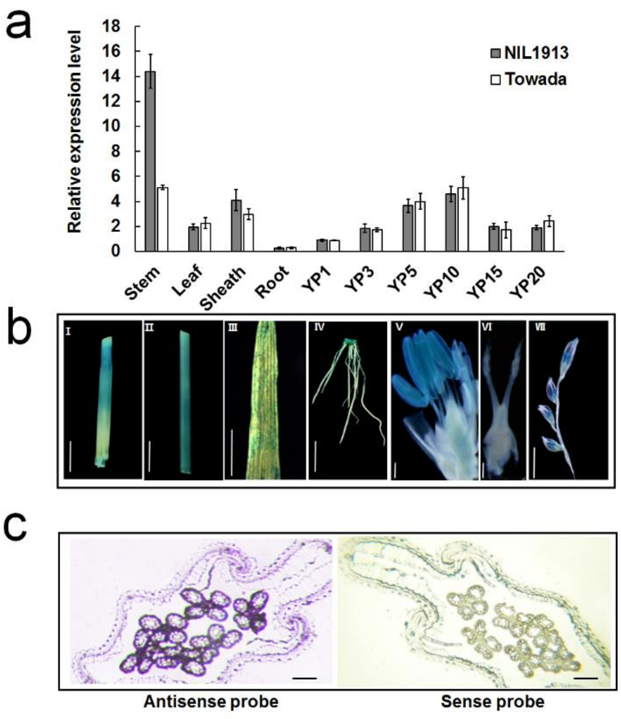

Supplementary Figure 17. Expression pattern analysis of *CTB4*. (a) qRT-PCR analysis of the expression levels of *CTB4a* in different tissues of NIL1913 and Towada. YP, young panicles, the numbers indicate the lengths of sampled young panicles. Data represent means ± s.d. (n=3). (**b**) GUS staining of different tissues using transgenic plants containing pCTB4a^{KMXBG}::GUS vector. Ⅰ, stem. Ⅱ, leaf sheath. Ⅲ, leaf. Ⅳ, root. Ⅴ, anther. Ⅵ, pistil. Ⅶ, spikelet. Scale bar, 1 cm in Ⅰ, Ⅱ, Ⅲ and Ⅳ. Scale bars, 0.5 mm in Ⅴ and Ⅵ, 0.5 cm in Ⅶ. (**c**) *In situ* hybridization of *CTB4a* in NIL1913 at stgae10. Scale bars, 100 μm.

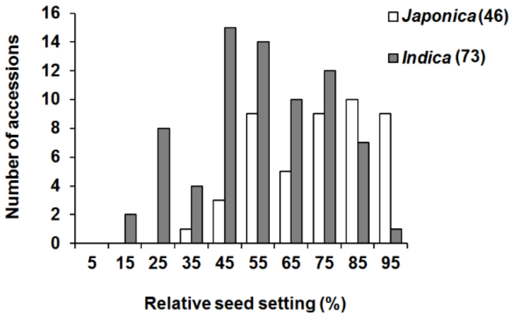

Supplementary Figure 18. Phenotypic distribution of *japonica* and *indica* subpopulations under cold stress at the booting stage.

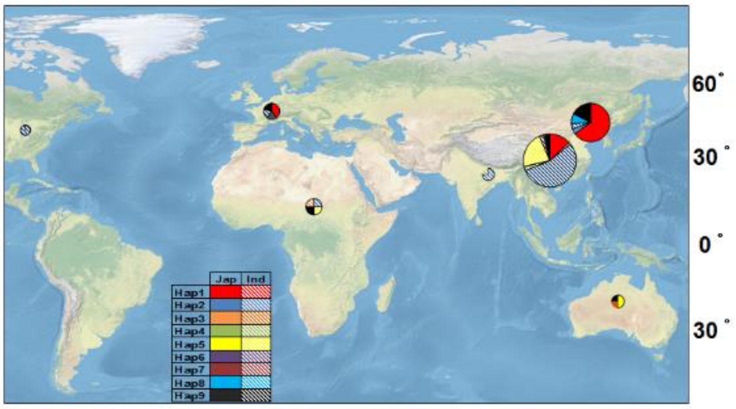

Supplementary Figure 19. Geographic distribution of nine haplotypes. For each haplotype, *japonica* and *indica* varieties were represented by solid and lattice colors respectively. The origins of the rice accessions were divided into several regions including south China, north China, south Asia, Europe, Africa, America and Oceania.

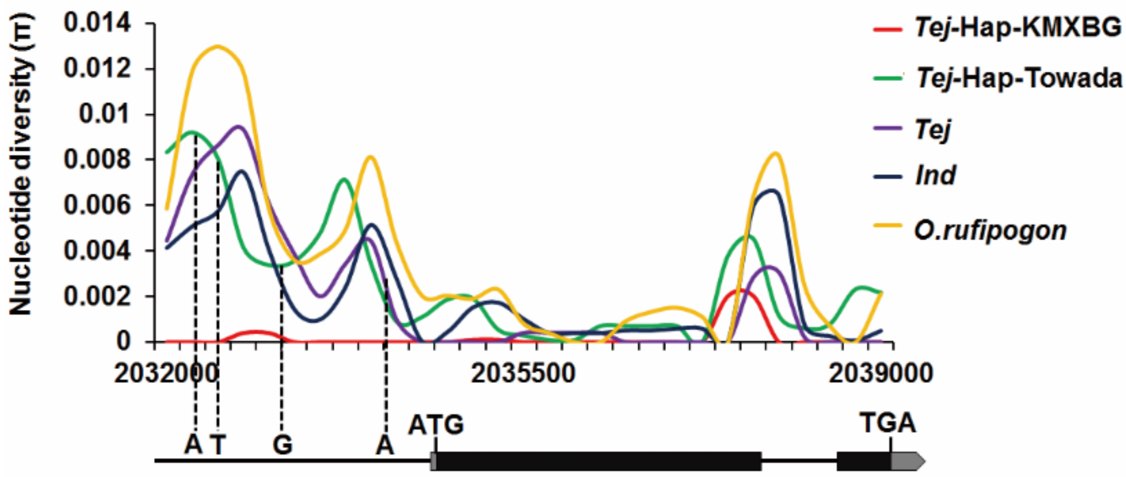

Supplementary Figure 20. Selective sweep signals in *CTB4a*. The Y-axis indicates π values. The four SNPs in the promoter region of *CTB4a* are denoted by black dotted lines.

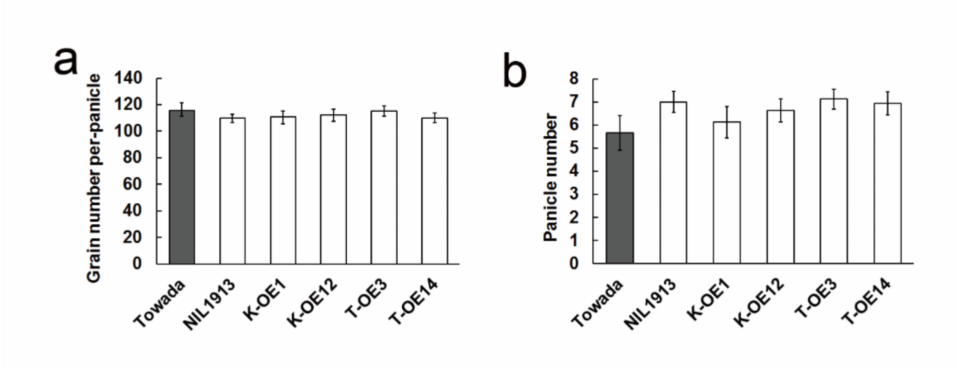

Supplementary Figure 21. Yield traits of *CTB4a* overexpression lines under CS-HAA. (**a**) Grain number per-panicle and (**b**) panicle number of Towada, NIL1913 and *CTB4a* overexpression lines grown under CS-HAA. Data represent means ± s.d. (n=30) and P values were calculated by Student's t tests. ** P < 0.01, * P < 0.05.

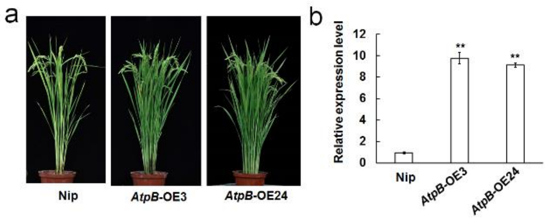

Supplementary Figure 22. *AtpB* overexpression lines. (a) Phenotype of Nip and *AtpB* overexpression lines. (b) Expression level of *AtpB* in overexpression lines. Three independent RNA samples were prepared and each experiment was performed with three technical repeats. Data represent means ± s.d. **P < 0.01, Student's t test.

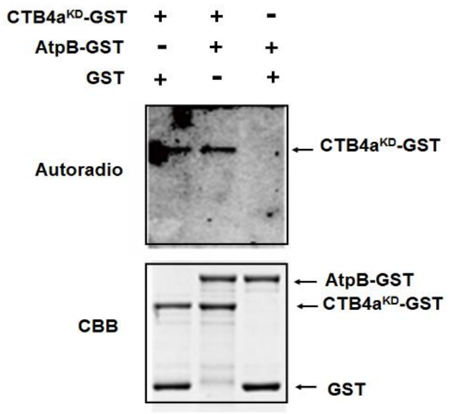

Supplementary Figure 23. Phosphorylation assay in *vitro*. Shown is auto-radiographed for 24 h (top panel) and SDS-PAGE gel with coomassie brilliant blue stained proteins (bottom panel).

.

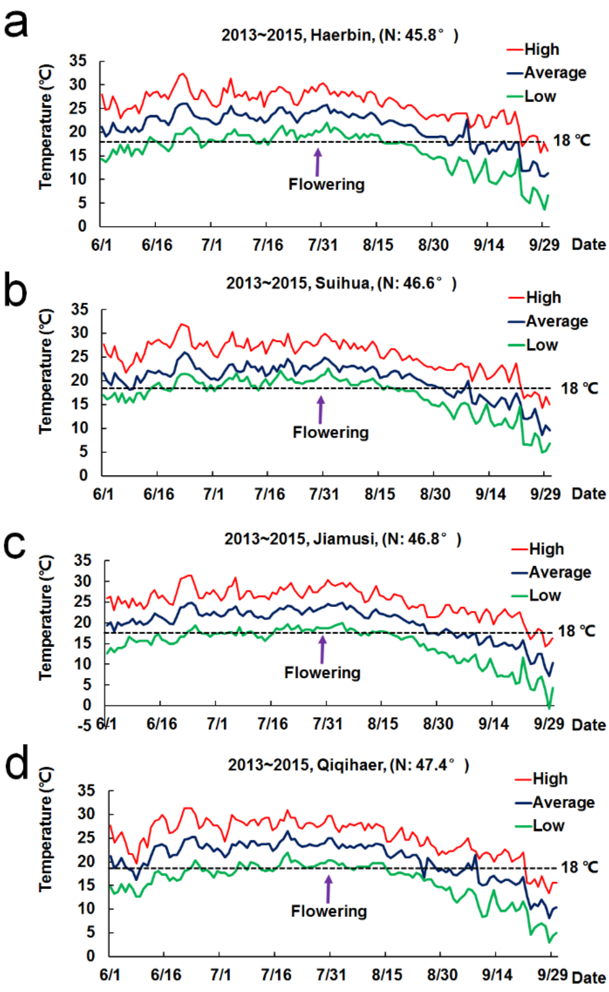

Supplementary Figure 24. Average temperature of four cities in Heilongjiang province in the summers of 2013 to 2015. Weather records of (**a**) Haerbin, (**b**) Suihua, (**c**) Jiamusi and (**d**) Qiqihaer were obtained from the local weather station. These places are the main rice producing areas in Heilongjiang province and were also called as cold rice growing zone.

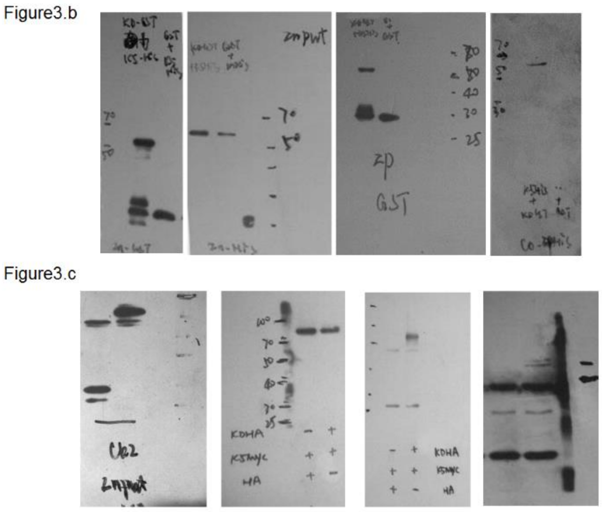

Supplementary Figure 25. The original western blot images used in this study.

Supplementary Table 1. Agronomic traits of NIL1913 and Towada under normal and CS-HAA conditions

Traits	Normal conditions (Beijing)		CS-HAA (Kunming)	
	Towada	NIL1913	Towada	NIL1913
Plant height (cm)	115.40±3.78	117.62±4.28	72.8±1.89	82.3±3.35**
Flag leaf length (cm)	41.30±3.95	38.21±7.46	24.23±5.43	26.7±5.16
Flag leaf width (cm)	1.60±0.09	1.40±0.08	1.28±0.03	1.35±0.04
Panicle length (cm)	21.28±2.91	22.16±1.21	17.00±0.50	18.46±0.57*
1st Inter-node length (cm)	37.40±1.92	40.26±2.47	28.03±0.68	31.15±1.80
Panicle exsertion (cm)	10.20±1.57	11.74±1.70	4.13±0.50	6.83±0.90*
Full grains per-panicle	126.00±16.07	136.80±19.38	56.33±16.56	96.20±13.42**
Total grains per panicle	135.00±16.48	149.2±29.51	125.00±13.75	153.80±17.46
Mean spikelet fertility (%)	93.94±2.81	92.55±5.59	44.90±0.09	62.86±0.08**

+/- indicates standard error, n = 15， ** P < 0.01, * P < 0.05，student's t-test.

Supplemental Table 2. Nucleotide polymorphism and neutrality tests of *CTB4a*

Taxon	N	L	S	H	π	Tajima's *D*	MLHKA
All-*Tej*	115	7092	135	12	0.00351	-1.24 P > 0.100	0.08700
Tej-Hap-KMXBG	36	7092	64	3	0.00018	-2.85 P < 0.001	3.1E-05
Tej-Hap-Towada	79	7092	95	8	0.00266	-0.45 P > 0.100	0.098
Trj	18	7092	82	3	0.00271	-0.99 P > 0.100	0.088
Ind	194	7092	69	11	0.00223	-0.52 P > 0.100	0.126
O.rufipogon	10	7092	105	8	0.00413	-0.24 P > 0.100	-

N, total number of samples. L, averages length (bp) of the sequences. S, number of polymorphic (segregating) sites. H, number of haplotypes. π, average number of pairwise nucleotide differences per site. MLHKA, the *P-values* of the maximum likelihood Hudson–Kreitman–Aguadé (MLHKA) test that was performed with *O. barthii* as the outgroup and seven neutrally evolving rice genes as the reference.

Supplementary Table 3. Average nucleotide diversity of *CTB4a* and 20 kb flanking region.

Loci	*Tej*			*Trj*	*Ind*	*O.rufipogon*
	All	Hap-KMXBG	Hap-Towada			
Upstream 20kb	0.00555	0.0008	0.00513	0.00272	0.00384	0.00661
CTB4a	0.00351	0.00018	0.00266	0.00271	0.00223	0.00413
Downstream 20kb	0.00445	0.00154	0.00436	0.00297	0.00288	0.00521

Supplementary Table 4. CTB4a interacting proteins isolated by yeast two-hybrid assay.

No.	ID	Annotation
1（AtpB）	LOC_ Os05g47980	ATP synthase subunit beta, putative, expressed
2	LOC_Os01g49190	ATP synthase subunit beta, putative, expressed
3	LOC_Os03g57790	Ubiquitin-conjugating enzyme, putative, expressed
